# No causal relationship between serum vitamin D levels and alcoholic liver disease: a two-sample bidirectional Mendelian randomization study

**DOI:** 10.3389/fnut.2024.1292954

**Published:** 2024-07-31

**Authors:** Huan Wu, Long Wu, Quan Zhang, Can Li, Hai-yang Li, Bao-fang Zhang

**Affiliations:** ^1^Department of Infectious Diseases, The Affiliated Hospital of Guizhou Medical University, Guiyang, Guizhou, China; ^2^Department of Anus and Intestinal Surgery, The Affiliated Hospital of Guizhou Medical University, Guiyang, Guizhou, China; ^3^Department of Hepatobiliary Surgery, The Affiliated Hospital of Guizhou Medical University, Guiyang, Guizhou, China

**Keywords:** vitamin D, alcoholic liver disease, GWAS, Mendelian randomization, SNPS

## Abstract

**Background:**

Numerous observational studies have presented an association between Vitamin D (VD) and Alcoholic Liver Disease (ALD). However, sufficient evidence from Randomized Controlled Trials (RCTs) substantiating this correlation is scarce, thus leaving the causality of this relationship ambiguous. To overcome the shortcomings of traditional observational studies, we performed a two-sample bidirectional Mendelian randomization (MR) analysis to ascertain the causal relationship between VD and ALD.

**Methods:**

We utilized summary statistics datasets from Genome-Wide Association Studies (GWAS) for VD and ALD. We selected genetic instruments that measure circulating VD levels (*n* = 64,979), and retrieved ALD statistics from GWASs, inclusive of 1,416 cases and 217,376 healthy controls, while excluding chronic liver diseases such as nonalcoholic fatty liver disease, toxic liver disease, and viral hepatitis. Subsequent, MR analyses were performed to obtain effect estimates using inverse variance weighted (IVW) random effect models. Cochran’s Q statistic and MR-Egger regression intercept analyses were used to assess pleiotropy. Sensitivity analyses using the MR Egger, weighted median, simple mode, and weighted mode methods were also performed. Leave-one-out analysis was used to identify SNPs with potential effect. Reverse MR analysis was also performed.

**Results:**

In IVW, our MR analysis incorporated 21 independent SNPs, circulating VD levels had no causal effect on ALD [*OR* = 0.624 (0.336–1.160), *p* = 0.136] and ALD had no causal effect on circulating VD [*OR* = 0.997 (0.986–1.008), *p* = 0.555]. No heterogeneity or pleiotropy was observed (*p* > 0.05). Other MR methods also agreed with IVW results.

**Conclusion:**

This study provides the causal relationship between genetically predicted circulating Vitamin D levels and ALD and provides new insights into the genetics of ALD.

## Introduction

Alcoholic Liver Disease (ALD) stands out as a significant global contributor to liver-related disorders, stemming from the detrimental impact of prolonged and excessive alcohol consumption. Notably, recent years have witnessed a consistent rise in the prevalence of alcohol consumption and alcohol addiction on a global scale. This surge has consequently been linked to an escalated overall mortality rate, thus further accentuating the substantial clinical and socioeconomic burden associated with ALD. Nevertheless, within this intricate landscape, the interplay between ALD, genetic susceptibility, and environmental influences remains multifaceted. The deficiency of key dietary nutrients, VD being a pertinent example, has been identified as a potential factor in the initiation of ALD (1, 2).

Vitamin D is produced by the skin upon exposure to sunlight and are also obtained through the diet, exhibits diverse physiological functions. Post synthesis or ingestion, these hormones undergo hydroxylation in the liver to form 25-hydroxyvitamin D [25(OH)D], which is the main circulating form of VD3 in humans. VD not only affects bone and calcium metabolism function, but also reduces the risk of chronic diseases, which including diabetes, cancer, cardiovascular, infectious, and auto-immune diseases (3). In recent years, in the mechanisms of liver inflammation and injury induced by ethanol, the dysfunction of anti-inflammatory and antioxidant functions may exacerbate the progression of the disease. The anti-inflammatory and antioxidant effects of VD have become a focal point of research, particularly concerning its role in the occurrence and development of chronic liver diseases (4, 5). Accumulated observational studies in humans show an inverse relationship between VD levels, and the risk and severity of ALD (2, 4, 6). Despite several studies reporting decreased VD levels in ALD patients and suggesting potential benefits of enhancing VD levels, the effect of VD supplementation in these patients remains debatable (7). Establishing causality remains challenging due to potential confounders and reverse causation.

Mendelian randomization is an epidemiological tool used for establishing causal inference between exposure and outcomes by employing genetic variation as instrumental variables (IVs). This allows MR to overcome the limitations of traditional observational studies and significantly eliminating reverse causation (8). Nonetheless, to date, there have been no MR studies to investigate the causal relationship between serum VD levels and ALD.

The definition of outcome and selection of instrumental variables critically influence MR findings. This study aimed to assess the risk of ALD in the United Kingdom Biobank (UKBB) cohort by conducting GWAS with a broader case definition than employed in previous works. Subsequently, utilizing genetic instruments derived from a European population’s meta-analysis GWAS on VD status, we conducted a two-sample bidirectional MR analysis to estimate first the effect of genetically predicted serum VD levels on risk of ALD, and reciprocally to estimate the causal effect of genetic risk for ALD on serum VD levels.

## Materials and methods

**Figure 1:**
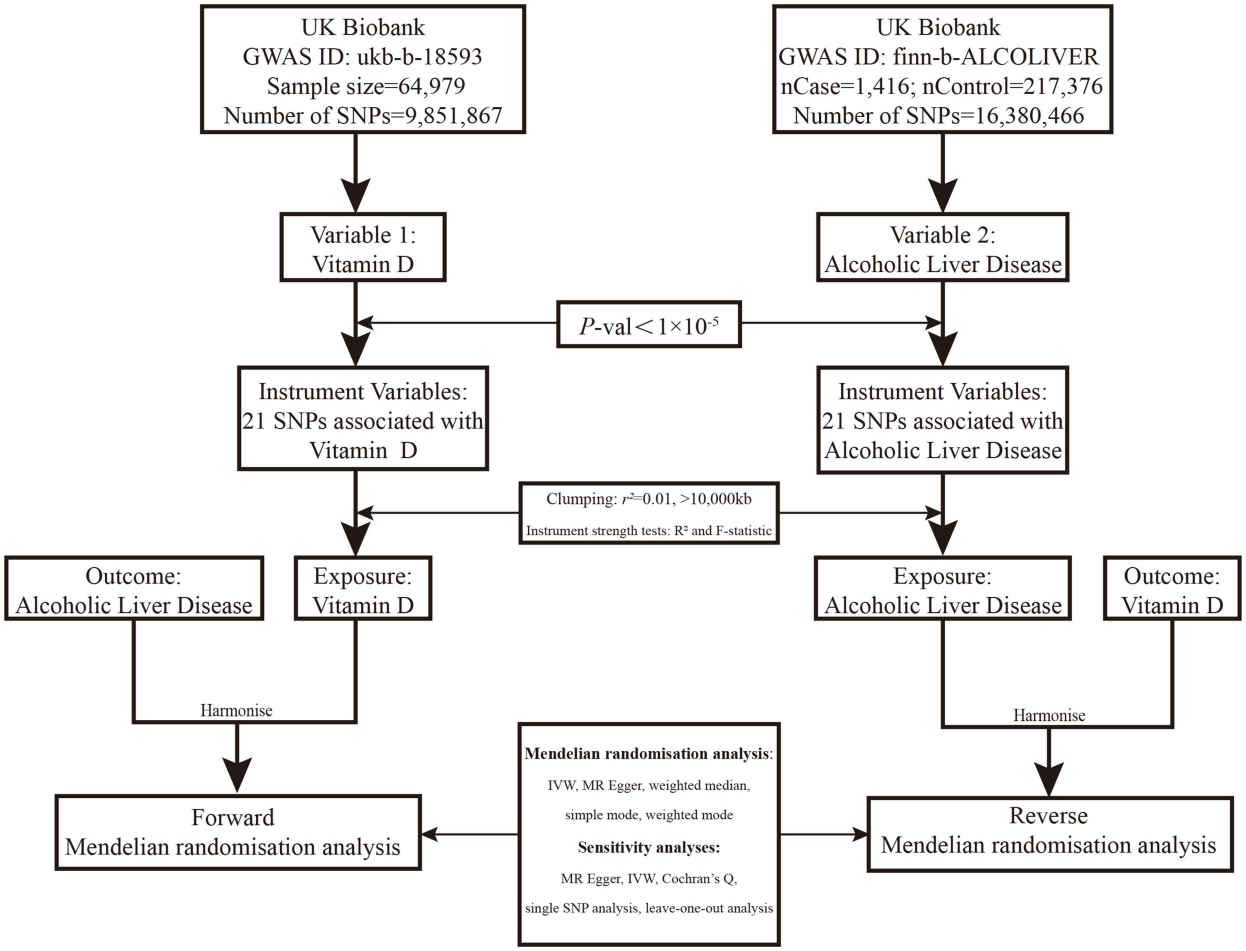
Overview of the two-sample MR study design used to investigate the probability of bidirectional association between serum VD and ALD. VD, Vitamin D; GWAS, Genome-wide association study; IVW, Inverse-variance weighted; MR, Mendelian randomization; ALD, Alcoholic liver disease; and SNP, Single-nucleotide polymorphism. SNPs were included if identified to be associated with ALD in the Speliotes’ GWAS with effects in the same direction with a *p* value of <1*10^−5^ in UKBB or were “discovered” in our GWAS in UKBB with a *p* value of <1*10^−5^.

The design of this two-sample bidirectional MR is summarized conclusion, which illustrated in Figure 1.

### Software selection and data source

We conducted our search on the MR Base database,^1^ a repository containing a substantial number of summary statistic data from hundreds of GWASs (9). To mitigate any potential bias that might arise due to population stratification, we only included subjects of European genetic origin in our study. The summary statistics datasets for VD, publicly available and derived from GWAS meta-analyses concerning European individuals (*n* = 64,979; GWAS ID: ukb-b-18593), served as our exposure. We sourced the ALD dataset from the most extensive histology-based ALD GWAS, comprising 1,416 European ALD cases and 217,376 genetically matched controls (GWAS ID: finn-b-ALCOLIVER). Each dataset was obtained from the published summarized results of publicly available, genome-wide association studies.

### Selection of the genetic instrument

The effect of VD levels on the risk of ALD (Variable 1, Figure 1) was evaluated using SNPs discovered in the Study of Underlying Genetic Determinants of VD, which showed an association with VD status. These SNPs were utilized as IVs to examine the correlation between genetically-inferred serum VD levels and ALD risk in the UKBB population cohort. A two-sample MR study of genetic variants linked with VD was employed as the IV, refining inference based on a *p* value threshold of 1*10^−5^ to capture as many potential genetic variants as possible. We procured summary statistics, including beta coefficients and standard errors, for 21 SNPs associated with VD, using these as IVs based on data from GWASs on VD. Conversely, we investigated the effect of ALD risk on VD levels using data from GWAS on ALD, which identified 21 standalone, genome-wide significant SNPs (Variable 2, Figure 1).

### Statistical analysis for Mendelian randomization

Mendelian randomization analysis is a statistical technique that requires genetic variants to be related to the exposure of interest, but not potential confounders, to establish causal relationships (10). Our study followed a three-step approach to investigate the association between VD and the risk of ALD. Firstly, we examined the independent association of SNPs with VD levels and the risk of ALD. Secondly, we assessed the association between each SNP and the risk of ALD. At the same time, we assessed the association between each SNP and VD levels. Lastly, we utilized two-sample bidirectional MR analysis, a method that leverages summary statistics from different GWASs (11), to estimate the causal relationship between VD and the risk of ALD. For this analysis, we employed 21 SNPs as IVs obtained from the VD and ALD GWASs. By using this MR approach, we aimed to derive an unbiased estimate of the causal association between VD and the risk of ALD, while minimizing the influence of confounding factors (Table 1).

**Table 1:** The results of heterogeneity and sensitivity test.

	Methods	*Q*	df	Q-val	*I* ^2^
Heterogeneity test	Vitamin D on ALD				
	MR Egger	19.076	19	0.452	0.004
	Inverse variance weighted	19.327	20	0.501	0.035
	ALD on Vitamin D				
	MR Egger	16.054	19	0.654	0.184
	Inverse variance weighted	17.670	20	0.610	0.132
Sensitivity test	Egger regression intercept	Standard error		Directionality *p* value	
	Vitamin D on ALD				
	0.016	0.032		0.623	
	ALD on Vitamin D				
	0.006	0.005		0.219	

In this study, the IVW method amalgamates the Wald ratio estimates of the causal effect procured from multiple genetic variants, lending a consistent estimation of the causal effect of the exposure variable on the outcome (12). To tackle potential pleiotropy, where genetic variants might influence multiple variables, we incorporated two additional methods: MR-Egger regression and the weighted median estimator. MR-Egger regression addresses unbalanced pleiotropy, factoring in a parameter for bias, via the use of summary data estimates of causal effects from each genetic variant (13). It executes a weighted linear regression of gene-outcome coefficients on gene-exposure coefficients, wherein the slope embodies the causal effect estimate. The average horizontal pleiotropic effect across genetic variants is estimated by the intercept (14). The weighted median estimator, on the other hand, provides a consistent estimate of the causal effect even if up to 50% of the information comes from genetic variants that are not valid instrumental variables (15). Sensitivity analyses using the MR Egger, weighted median, simple mode, and weighted mode methods were also performed (13, 15, 16). As each method makes slightly different assumptions, a consistent effect across multiple methods yields the most robust evidence of causal inference. A higher *R*^2^ and *F*-statistic denote a reduced risk of weak instrument bias, and hold greater precision in estimations compared to MR-Egger analyses. Statistical significance was set to *p* < 0.05. We ran all Mendelian randomization analyses on RStudio Software (Version: 2023.06.0 Build 421) and *R* Software (Version: 4.3.1) (Supplementary material 1).

### Heterogeneity and sensitivity test

We assessed the heterogeneities between SNPs using Cochran’s *Q*-statistics (17) band *I*^2^ statistic (18, 19). Additionally, we also conducted a “leave-one-out” analysis to explore the possibility of a causal association driven by a single SNP.

## Results

### Studies included in the meta-analysis

#### Instrumental variables for Mendelian randomization

In our analysis, we utilized a set of 21 independent SNPs identified from GWASs of VD and ALD as IVs. Each of these SNPs demonstrated a significant association with VD and ALD as per the genome-wide level of significance (refer to Supplementary Table S3 for more details). Of note, the *F* statistic, denoting the robustness of the IVs, was recorded to be 10 or above for each individual SNP variant. Conventionally, an *F* statistic under 10 typically signifies a “weak IV,” indicating that our study had minimal risk of weak instrument bias. The *F* statistic for all the SNPs used in the MR analysis was >10, verifying them as “strong” instruments. The *F* statistic measures the magnitude and precision of each SNP’s influence over VD and ALD. The individual *F* statistic for VD ranged between 20 and 23 (refer Supplementary Table S1) and for ALD, it ranged between 20 and 85 (refer Supplementary Table S2).

#### Mendelian randomization results

**Figure 2:**
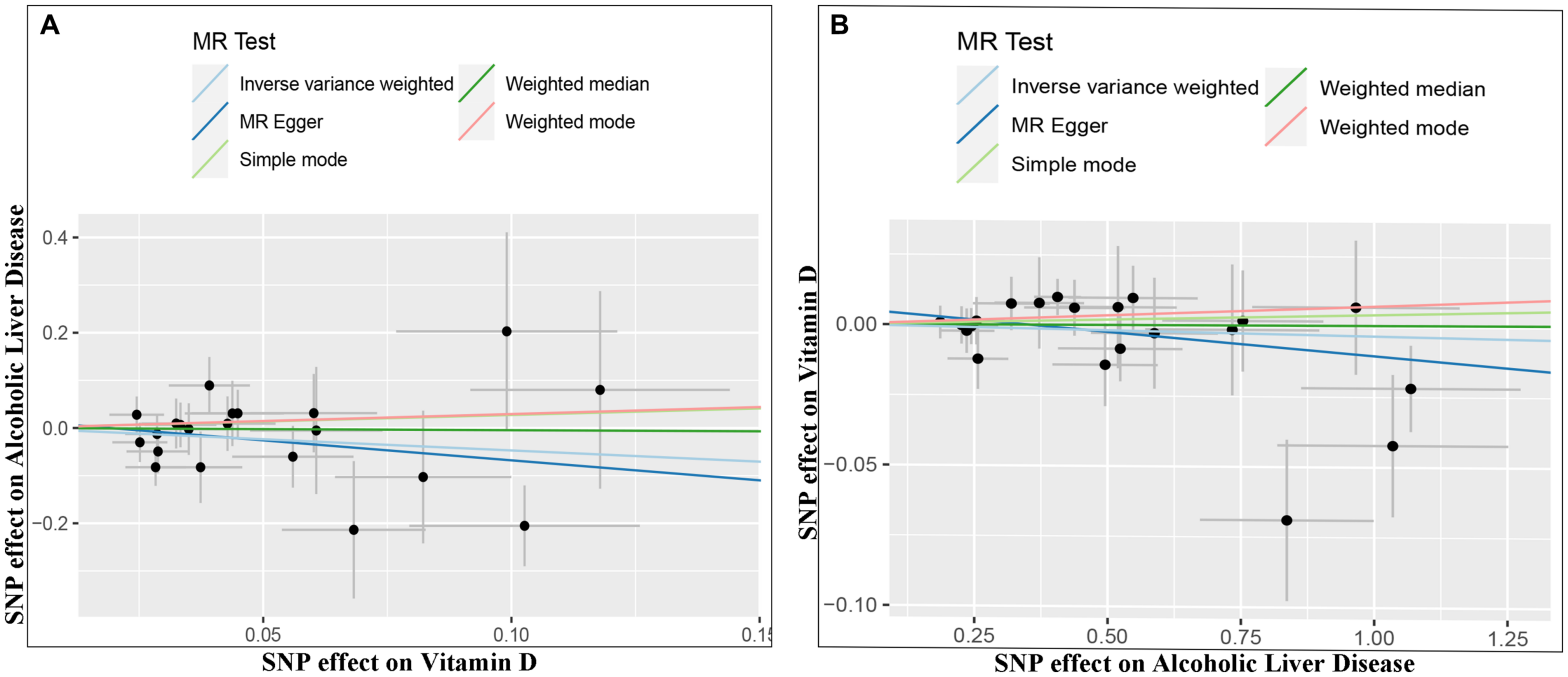
Scatter plots of genetic associations between Vitamin D and ALD. The slopes of each line represent the causal association for each method. The light blue line represents the inverse-variance weighted estimate, the green line represents the weighted median estimate, the dark blue line represents the Mendelian randomization-Egger estimate, the red line represents the weighted mode estimate, and the light green line represents the simple mode estimate (**A**, Effect of Vitamin D on ALD; **B**, Effect of ALD on Vitamin D).

**Figure 3:**
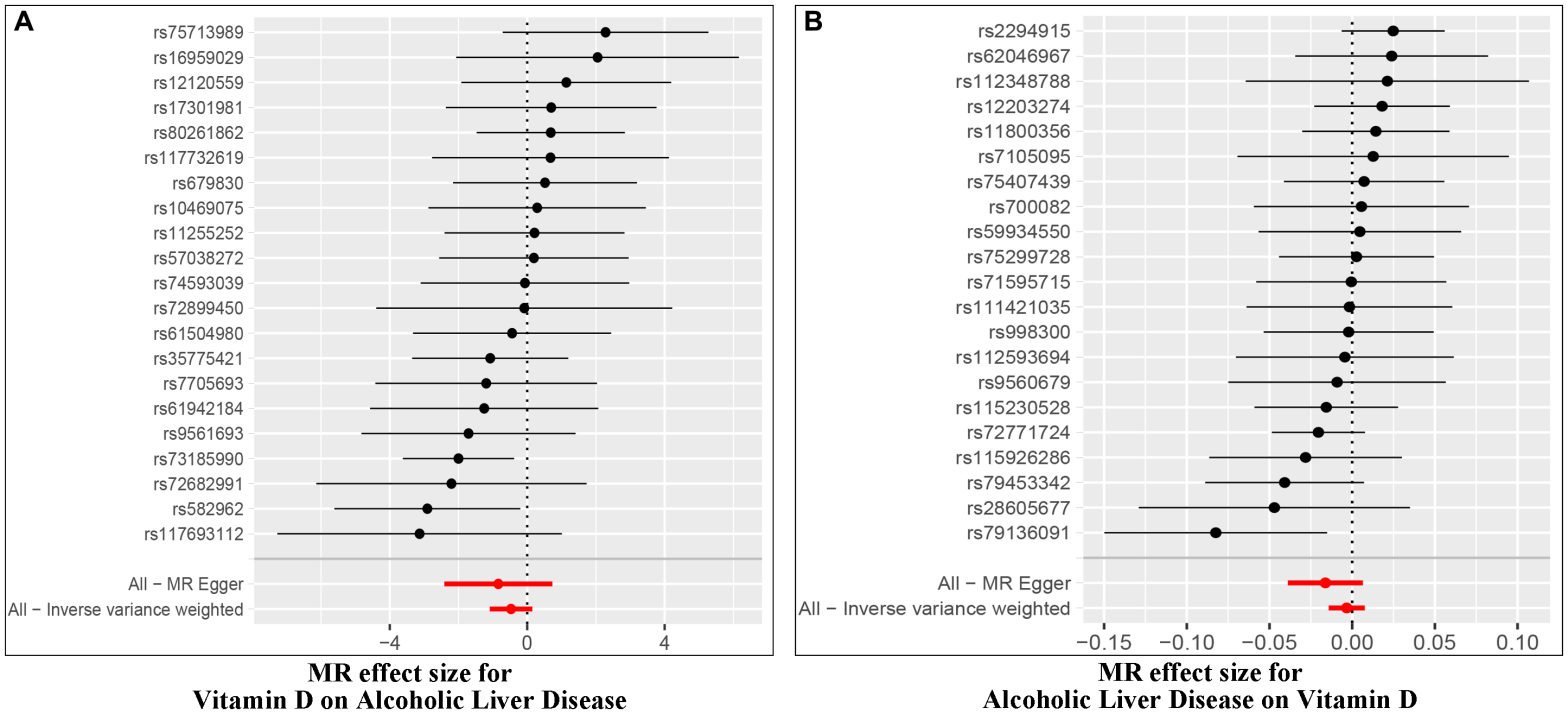
Forest plot of MR effect of the causal relationship between Vitamin D and ALD (**A**, Effect of Vitamin D on ALD; **B**, Effect of ALD on Vitamin D).

The IVW random method revealed no significant effect of serum VD levels on the risk of ALD, with an OR of 0.624 (0.336, 1.160) and a *p* value of 0.136 (see Table 2 and Figures 2, 3). Conversely, the IVW random effect analysis also found no evidence of a causal effect of ALD on the odds of VD, with an OR of 0.997 (0.986, 1.008) and a *p* value of 0.555 (consult Table 2 and Figures 2, 3). The intercept of the MR-Egger test, representing the mean pleiotropic effect across genetic variants, was insignificantly different from zero, suggesting that directional pleiotropy is unlikely to bias the results (refer to Table 1). Furthermore, an evaluation involving the MR-Egger analysis, weighted median, weighted mode, and simple mode found no causal association between VD and ALD.

**Table 2:** Results of two-sample bidirectional MR analysis of the causal effects between Vitamin D and ALD.

Exposures	Outcomes	Methods	Number of SNPs	Beta	SE	*p* value	OR	95%CI
Vitamin D on ALD								
Vitamin D	ALD	MR Egger	21	−0.839	0.802	0.308	0.432	0.090–2.081
		Weighted median	21	−0.046	0.466	0.921	0.955	0.383–2.381
		Inverse variance weighted	21	−0.471	0.316	0.136	0.624	0.336–1.160
		Simple mode	21	0.274	0.888	0.761	1.315	0.231–7.502
		Weighted mode	21	0.293	0.876	0.741	1.341	0.241–7.465
ALD on Vitamin D								
ALD	Vitamin D	MR Egger	21	−0.016	0.012	0.178	0.984	0.962–1.007
		Weighted median	21	0.000	0.008	0.955	1.000	0.984–1.017
		Inverse variance weighted	21	−0.003	0.006	0.555	0.997	0.986–1.008
		Simple mode	21	0.004	0.015	0.774	1.004	0.976–1.034
		Weighted mode	21	0.007	0.016	0.656	1.007	0.976–1.040

### Heterogeneity and sensitivity test

**Figure 4:**
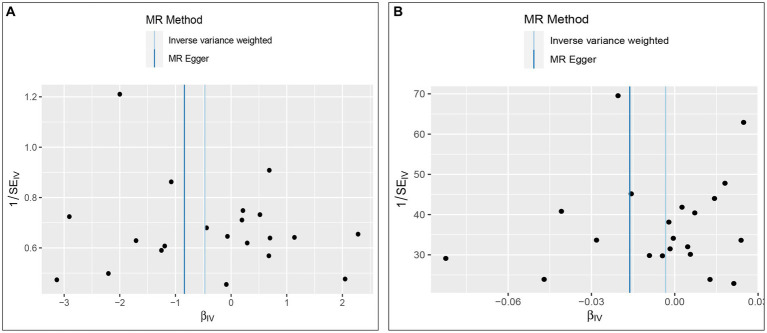
Funnel plot to assess heterogeneity. The light blue line represents the inverse-variance weighted estimate, and the dark blue line represents the Mendelian randomization-Egger estimate (**A**, Effect of Vitamin D on ALD; **B**, Effect of ALD on Vitamin D).

**Figure 5:**
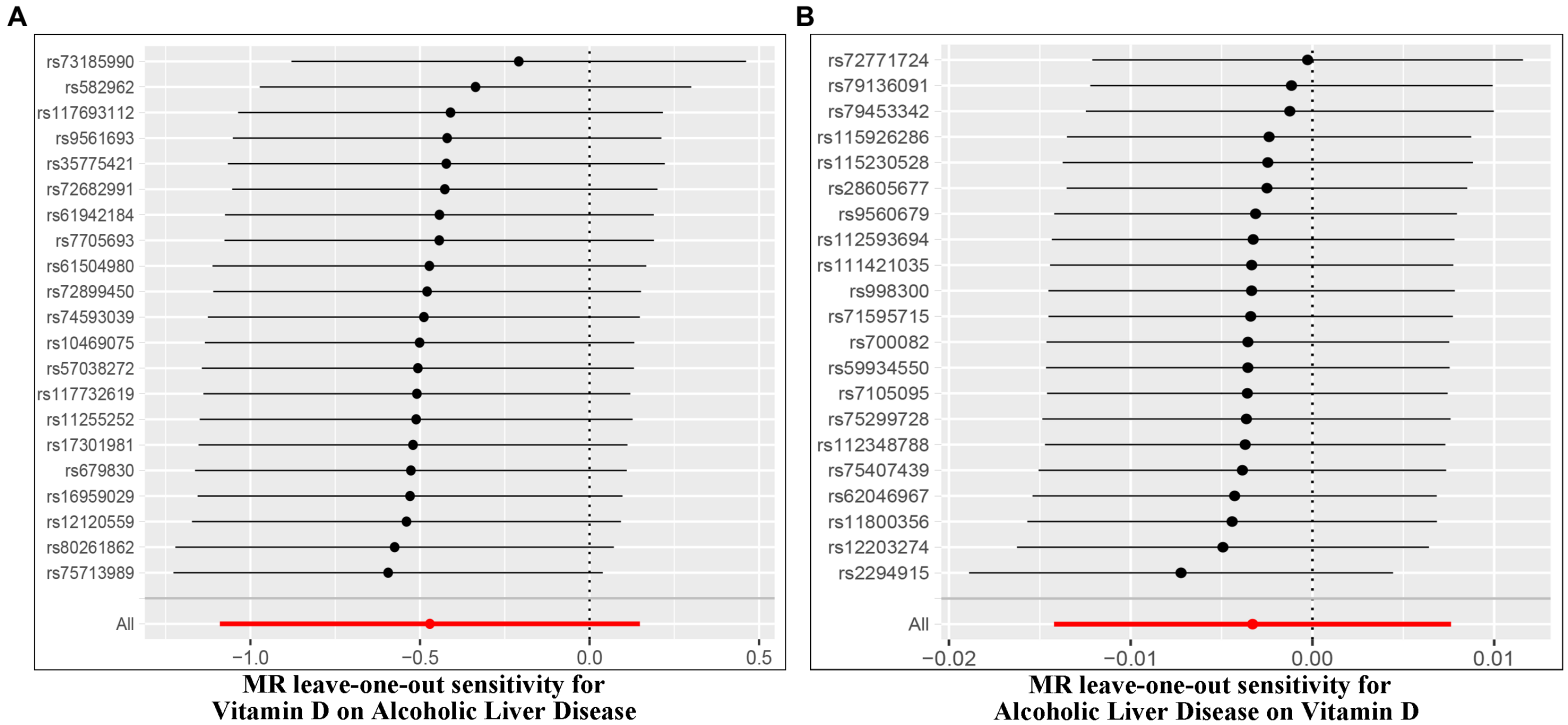
Leave-one-out of SNPs associated with Vitamin D and ALD. Each black point represents result of the IVW MR method applied to estimate the causal effect between Vitamin D and ALD [**(A)** Effect of Vitamin D on ALD; **(B)** Effect of ALD on Vitamin D].

The Cochran’s *Q* test was conducted to evaluate heterogeneity among instrumental variable estimates from individual genetic variants. The results did not indicate any substantial evidence of heterogeneity (refer to Table 1 and Figure 4). Heterogeneity is the variability in causal estimates obtained from each SNP. A low heterogeneity value signifies an increase in the reliability of MR estimates. Further strengthening the reliability of MR estimates, the *I*^2^ values also displayed low heterogeneity (refer to Table 1). In the “leave-one-out” analysis, each SNP was separately excluded to analyze its effect on the overarching IVW point estimate (refer to Figure 5). It was revealed that no single SNP had a significant impact on the IVW point estimate. This suggests that the cumulative result was not skewed by any particular genetic variant. A funnel plot was used to evaluate publication bias and directional horizontal pleiotropy, and it did not display any significant asymmetry. Furthermore, the MR-Egger regression test did not show any evidence of asymmetry, thereby further asserting the absence of bias due to directional horizontal pleiotropy (refer to Figure 4). Conclusively, the lack of considerable heterogeneity, low *I*^2^ values, outcomes of the “leave-one-out” analysis, and absence of asymmetry in the funnel plot and MR-Egger regression test affirm the reliability of the MR estimates and reduce apprehensions about potential analysis biases.

## Discussion

Existing studies have noted a link between serum VD levels and ALD (1, 2, 6). However, it is still uncertain whether this association is causal or the direction of the causality. In this extensive two-sample bidirectional MR study exploring the ViD and ALD relationship, we found no tangible evidence supporting a reciprocal causal link between serum VD levels and ALD susceptibility in a large cohort of European ancestry. To the best of our knowledge, this is the inaugural two-sample MR examination inspecting the link between serum VD levels and the risk of ALD within a European population.

Vitamin D deficiency is a widespread condition that has reached epidemic proportions in Western countries (20), primarily due to current lifestyle and limited dietary sources. An estimated billion people are deficient in VD (21). With anti-inflammatory, immunomodulatory, proapoptotic, and antiangiogenic effects, VD operates critical roles within the body (22–24), including protection against rickets/bone demineralization, hypertension, tumor, the body’s defense against infections, and autoimmune (25, 26). A damaging effect of VD insufficiency on the immune system could happen during severe chronic liver diseases (3). Besides, VD is involved in regulating adipose tissue inflammation, liver fibrosis, and predicting antiviral effects (27–30). It also influences insulin resistance and abnormal fat accumulation in the liver (24, 31, 32). Recently, reports have indicated VD deficiency in chronic liver diseases regardless of the etiology (33).

There is significant scientific interest in the connection between VD status and ALD. Observational studies have observed decreased VD levels in ALD (34). VD deficiency is a contributing factor to ALD (6). For instance, VD was an independent cofactor linked with the occurrence of ASH in alcoholic patients, who frequently had severe VD deficiency and bridging fibrosis (2). Its mode of action might be connected to the activation of the NF-κB signaling pathway, which is associated with promoting the inflammatory response (35). A relationship between severe VD deficiency exists and the mortality rate in alcoholic cirrhotic patients (4, 31). Consequently, VD has been regarded as a risk aspect for the evolution of ALD. Furthermore, SNPs within the VD receptor (VDR) gene have a recognized association with chronic liver disease (36, 37). In a study conducted by Saberi et al. (38), they proposed that the VDR activation signal interferes with the transforming growth factor beta-dependent transcriptional responses in profibrotic genes in HSCs. The VDR agonist calcifertriol reduces liver fibrosis in a mouse model of liver injury. Yet, as several ALD patients do not exhibit low VD levels, questioning the causality of VD ensues. While a large body of preclinical and observational data proposes a relationship between VD status and risk and severity of ALD (6), solid evidence from clinical intervention trials remains insufficient (39). A Cochrane review focusing on chronic liver disease in adults concluded that the current evidence does not support the use of VD supplements for the prevention or treatment of these conditions (7). Thus, it remains unclear whether VD has a causal association with ALD.

The MR methodology uses genetic variants linked with a modifiable exposure or biological intermediate to estimate the causal relationship between these variables and a medically pertinent outcome (40). MR circumvents many constraints of conventional epidemiological studies. The random distribution of genetic variants at conception minimizes confounding from environmental factors, thereby fortifying causal inference (41). MR analyses lessen confounding and reverse causality due to the parental random allocation of genotypes to offspring (42). To date, our current MR study is the first two-sample bidirectional MR evaluation to assess the causal role of VD for ALD in the European population. It merits mentioning that total serum VD incorporates VD bound to the VD binding protein (approximately 85%), VD bound to albumin (about 15%), and the fraction of free VD (less than 1%) (43). Contemporary studies have suggested that free or active serum VD may be a superior indicator of VD status compared with total serum VD. This is especially applicable in conditions like pregnancy, liver disease, or kidney disease, which influence VD binding protein levels (43, 44). Therefore, free VD was used as the phenotype in this study, and the genetic tools selected covered genetic variants associated with free serum VD. However, it is worth noting that general and clinical population-level correlations between free and total serum VD have mitigated this limitation to some extent (45). In this study, we executed five different estimation methods (inverse-variance weighted method, weighted median method, weighted mode, simple mode, and MR-Egger regression) for MR analyses. We applied the two-sample MR to assess the association between VD and ALD in this study. The findings indicated a lack of genetic evidence to conclusively support a causal relationship between VD levels and the risk of ALD. This conclusion remained unwavering even in the wake of sensitivity analyses and further replication. In addition, we further divided VD levels into high or low groups and conducted a Mendelian randomization study on the relationship between dichotomous VD levels and ALD risk. Since we did not find a GWAS dataset to obtain high VD levels as an exposure factor, and VD deficiency is more common in clinical practice. Therefore, we conducted a supplementary study focusing on the association between VD deficiency and ALD risk. Our results are consistent with the current study, indicating that there is no causal relationship between VD deficiency and increased ALD risk (see Supplementary material). This may provide additional perspectives on the relationship between VD levels and ALD risk.

Divergences between conclusions may be related to several reasons. On one hand, we tend to suggest that this discrepancy could hint at the flaw (residual confounding) of cross-sectional studies. The remaining association will often still be a biased estimate due to the existence of unknown or unmeasured confounders [sun exposure, physical activity, obesity, insulin resistance, different sample sizes, races, Body Mass Index (BMI), and so on], or imprecision in measured confounders (31). On the other hand, observational studies can be hindered by confounding or reverse causation (46). In addition, given the multifaceted roles of VD in the body, its causal relationship with ALD may be influenced by a variety of physiological and environmental factors. This could potentially explain our observed lack of significant results.

## Strengths and limitations

This research encompassed several strengths and limitations. Firstly, the exact function of certain SNPs remains unknown, potentially allowing residual bias when examining pleiotropy. However, we procured consistent results utilizing five MR methods considering pleiotropy, robust methodology, and sensitivity analyses that excluded SNPs with pleiotropic impacts, which is reassuring. Despite the fact that the SNPs used as instruments in our MR were extracted from GWAS in Europeans, the populations of both GWAS were not homogenous in terms of geographic location. It has been shown that there could be some gene–environment interaction in the effect of SNPs in the VD receptor gene on Chronic liver disease risk (36, 37). This raises a possibility of gene–environment interaction for SNPs affecting VD levels and of nonlinear effects of these SNPs on risk of ALD, but two-sample MR studies can only assess linear associations. Secondly, the study population consisted of Europeans, and there were differences in sample size between the VD and ALD datasets. Therefore, Our MR results cannot be generalized to non-Europeans and potentially to Europeans residing in different geographic areas than those of the participants in the VD and ALD GWAS (47). Due to ethnic differences in exposure and outcome GWAS populations, we also cannot completely rule out residual confounding by population stratification (46). We believe that potential biases can be avoided in the future by including more databases of people of non-European ancestry and increasing the sample size. Thirdly, owing to the unavailability of specifics about participant overlap between the two published GWAS summary datasets, it was not possible to compute potential biases arising from participant overlap. Last but not least, the use of two-sample Mendelian randomization enabled us to conduct the largest genome-wide association study on ALD yet undertaken, improving the likelihood of establishing a causal relationship between VD levels and ALD risk. There was less likelihood of confounding and reverse causality bias in this study than in previous routine observational studies.

The future course of this research encompasses broadening the MR strategy to populations beyond European descent. The work will investigate the potential alteration of genetically forecasted VD influences on ALD risk and severity subject to ALD risk factors. These potential aspects include race, ethnicity, age, sex, BMI, and prospective MR analyses, employing updated GWAS samples along with different cohorts.

## Conclusion

In conclusion, despite cross-sectional studies exhibiting an association between Vitamin D concentration and ALD in both mappings. Our study suggested that no causal relationship was found between Vitamin D deficiency and ALD; neither did Vitamin D deficiency pose a risk factor for the development of the disease. Negative results are not meaningless, and many current MR studies have broken the conclusions of observational studies (48, 49). In the future, there is a need for a larger sample size and GWAS data of non-European ancestry patients to update the conclusion.

## Data availability statement

Publicly available datasets were analyzed in this study. This data can be found at: https://gwas.mrcieu.ac.uk/.

## Ethics statement

Ethical approval was not required for the study involving humans in accordance with the local legislation and institutional requirements. Written informed consent to participate in this study was not required from the participants or the participants’ legal guardians/next of kin in accordance with the national legislation and the institutional requirements.

## Author contributions

HW: Conceptualization, Data curation, Investigation, Methodology, Project administration, Resources, Writing – review & editing. LW: Conceptualization, Data curation, Formal analysis, Funding acquisition, Methodology, Project administration, Writing – original draft, Writing – review & editing. QZ: Methodology, Project administration, Software, Supervision, Validation, Writing – original draft, Writing – review & editing. CL: Conceptualization, Investigation, Methodology, Validation, Writing – original draft, Writing – review & editing. H-yL: Conceptualization, Funding acquisition, Writing – review & editing. B-fZ: Conceptualization, Funding acquisition, Software, Supervision, Validation, Visualization, Writing – original draft, Writing – review & editing.

## Footnotes

^1^ http://www.mrbase.org/

## References

[1] Fisher L Fisher A. Vitamin D and parathyroid hormone in outpatients with noncholestatic chronic liver disease. Clin Gastroenterol Hepatol. (2007) 5:513–20. doi: 10.1016/j.cgh.2006.10.015, PMID: 17222588

[2] Anty R Canivet CM Patouraux S Ferrari-Panaia P Saint-Paul MC Huet PM. Severe vitamin D deficiency may be an additional cofactor for the occurrence of alcoholic steatohepatitis. Alcohol Clin Exp Res. (2015) 39:1027–33. doi: 10.1111/acer.12728, PMID: 25941109

[3] Iruzubieta P Teran A Crespo J Fabrega E. Vitamin D deficiency in chronic liver disease. World J Hepatol. (2014) 6:901–15. doi: 10.4254/wjh.v6.i12.901, PMID: 25544877 PMC4269909

[4] Trepo E Ouziel R Pradat P Momozawa Y Quertinmont E Gervy C. Marked 25-hydroxyvitamin D deficiency is associated with poor prognosis in patients with alcoholic liver disease. J Hepatol. (2013) 59:344–50. doi: 10.1016/j.jhep.2013.03.024, PMID: 23557869

[5] Li LH Yin XY Yao CY Zhu XC Wu XH. Serum 25-hydroxyvitamin D, parathyroid hormone, and their association with metabolic syndrome in Chinese. Endocrine. (2013) 44:465–72. doi: 10.1007/s12020-013-9885-2, PMID: 23340918

[6] Bingbing Y Chunqiu H Yongdi H Lanlan C Dexiang X. Associations between vitamin D deficiency and alcoholic liver disease. Acta Univ Med Anhui. (2019) 54:1273–6. doi: 10.19405/j.cnki.issn1000-1492.2019.08.021

[7] Bjelakovic M Nikolova D Bjelakovic G Gluud C. Vitamin D supplementation for chronic liver diseases in adults. Cochrane Database Syst Rev. (2021) 2021:CD011564. doi: 10.1002/14651858.CD011564.pub3, PMID: 34431511 PMC8407054

[8] Smith GD Ebrahim S. ‘Mendelian randomization’: can genetic epidemiology contribute to understanding environmental determinants of disease? Int J Epidemiol. (2003) 32:1–22. doi: 10.1093/ije/dyg070, PMID: 12689998

[9] Bae S-C Lee YH. Causal association between body mass index and risk of rheumatoid arth ritis: a Mendelian randomization study. Eur J Clin Investig. (2019) 49:e13076. doi: 10.1111/eci.13076, PMID: 30710354

[10] Burgess S Butterworth A Thompson SG. Mendelian randomization analysis with multiple genetic variants using summarized data. Genet Epidemiol. (2013) 37:658–65. doi: 10.1002/gepi.21758, PMID: 24114802 PMC4377079

[11] Hartwig FP Davies NM Hemani G Davey Smith G. Two-sample Mendelian randomization: avoiding the downsides of a powerful, widely applicable but potentially fallible technique. Int J Epidemiol. (2016) 45:1717–26. doi: 10.1093/ije/dyx028, PMID: 28338968 PMC5722032

[12] Pierce BL Burgess S. Efficient design for Mendelian randomization studies: subsample and 2-sample instrumental variable estimators. Am J Epidemiol. (2013) 178:1177–84. doi: 10.1093/aje/kwt084, PMID: 23863760 PMC3783091

[13] Bowden J Davey Smith G Burgess S. Mendelian randomization with invalid instruments: effect estimation and bias detection through Egger regression. Int J Epidemiol. (2015) 44:512–25. doi: 10.1093/ije/dyv080, PMID: 26050253 PMC4469799

[14] Burgess S Thompson SG. Interpreting findings from Mendelian randomization using the MR-Egger method. Eur J Epidemiol. (2017) 32:377–89. doi: 10.1007/s10654-017-0255-x, PMID: 28527048 PMC5506233

[15] Bowden J Davey Smith G Haycock PC Burgess S. Consistent estimation in Mendelian randomization with some invalid instruments using a weighted median estimator. Genet Epidemiol. (2016) 40:304–14. doi: 10.1002/gepi.21965, PMID: 27061298 PMC4849733

[16] Hartwig FP Davey Smith G Bowden J. Robust inference in summary data Mendelian randomization via the zero modal pleiotropy assumption. Int J Epidemiol. (2017) 46:1985–98. doi: 10.1093/ije/dyx102, PMID: 29040600 PMC5837715

[17] Egger M Smith GD Phillips AN. Meta-analysis: principles and procedures. BMJ. (1997) 315:1533–7. doi: 10.1136/bmj.315.7121.1533, PMID: 9432252 PMC2127925

[18] Bowden J Del Greco MF Minelli C Davey Smith G Sheehan NA Thompson JR. Assessing the suitability of summary data for two-sample Mendelian randomization analyses using MR-Egger regression: the role of the I2 statistic. Int J Epidemiol. (2016) 45:1961–74. doi: 10.1093/ije/dyw220, PMID: 27616674 PMC5446088

[19] Higgins JP Thompson SG. Quantifying heterogeneity in a meta-analysis. Stat Med. (2002) 21:1539–58. doi: 10.1002/sim.1186, PMID: 12111919

[20] Blachier M Leleu H Peck-Radosavljevic M Valla DC Roudot-Thoraval F. The burden of liver disease in Europe: a review of available epidemiological data. J Hepatol. (2013) 58:593–608. doi: 10.1016/j.jhep.2012.12.005 23419824

[21] Wacker M Holick MF. Vitamin D—effects on skeletal and extraskeletal health and the need for supplementation. Nutrients. (2013) 5:111–48. doi: 10.3390/nu5010111, PMID: 23306192 PMC3571641

[22] Adams JS Hewison M. Unexpected actions of vitamin D: new perspectives on the regulation of innate and adaptive immunity. Nat Clin Pract Endocrinol Metab. (2008) 4:80–90. doi: 10.1038/ncpendmet0716, PMID: 18212810 PMC2678245

[23] Monjour L Druilhe P Fribourg-Blanc A Karam M Froment A Feldmeier H. General considerations on endemic treponematosis in the rural Sahel region of upper Volta. Acta Trop. (1983) 40:375–82. PMID: 6142636

[24] Kitson MT Roberts SK. D-livering the message: the importance of vitamin D status in chronic liver disease. J Hepatol. (2012) 57:897–909. doi: 10.1016/j.jhep.2012.04.033, PMID: 22634121

[25] DeLuca HF. Overview of general physiologic features and functions of vitamin D. Am J Clin Nutr. (2004) 80:1689S–96S. doi: 10.1093/ajcn/80.6.1689S, PMID: 15585789

[26] Christakos S Dhawan P Porta A Mady LJ Seth T. Vitamin D and intestinal calcium absorption. Mol Cell Endocrinol. (2011) 347:25–9. doi: 10.1016/j.mce.2011.05.038, PMID: 21664413 PMC3405161

[27] Udomsinprasert W Jittikoon J. Vitamin D and liver fibrosis: molecular mechanisms and clinical studies. Biomed Pharmacother. (2019) 109:1351–60. doi: 10.1016/j.biopha.2018.10.140, PMID: 30551386

[28] Wang M Wang M Zhang R Shen C Zhang L Ding Y. Influences of vitamin D levels and vitamin D-binding protein polymorphisms on nonalcoholic fatty liver disease risk in a Chinese population. Ann Nutr Metab. (2022) 78:61–72. doi: 10.1159/000522193, PMID: 35100585 PMC9116593

[29] Marziou A Philouze C Couturier C Astier J Obert P Landrier JF. Vitamin D supplementation improves adipose tissue inflammation and reduces hepatic steatosis in obese C57BL/6J mice. Nutrients. (2020) 12:342. doi: 10.3390/nu12020342, PMID: 32012987 PMC7071313

[30] Abbas MA. Physiological functions of vitamin D in adipose tissue. J Steroid Biochem Mol Biol. (2017) 165:369–81. doi: 10.1016/j.jsbmb.2016.08.004 27520301

[31] Testino G Leone S Fagoonee S. Alcoholic liver disease and vitamin D deficiency. Minerva Med. (2018) 109:341–3. doi: 10.23736/S0026-4806.18.05732-4 29963832

[32] Falleti E Bitetto D Fabris C Fattovich G Cussigh A Cmet S. Vitamin D binding protein gene polymorphisms and baseline vitamin D levels as predictors of antiviral response in chronic hepatitis C. Hepatology. (2012) 56:1641–50. doi: 10.1002/hep.25848, PMID: 22610885

[33] Pop TL Sirbe C Benta G Mititelu A Grama A. The role of vitamin D and vitamin D binding protein in chronic liver diseases. Int J Mol Sci. (2022) 23:10705. doi: 10.3390/ijms231810705, PMID: 36142636 PMC9503777

[34] Malham M Jorgensen SP Ott P Agnholt J Vilstrup H Borre M. Vitamin D deficiency in cirrhosis relates to liver dysfunction rather than aetiology. World J Gastroenterol. (2011) 17:922–5. doi: 10.3748/wjg.v17.i7.922, PMID: 21412501 PMC3051142

[35] Chun-qiu H De-xiang X Guo-xiu W Li L Su-fang W Chuan-lai H. Effects of vitamin d deficiency on alcohol-induced liver injury. Acta Nutr Sinica. (2020) 42:465–71. doi: 10.13325/j.cnki.acta.nutr.sin.2020.05.008

[36] Tourkochristou E Tsounis EP Tzoupis H Aggeletopoulou I Tsintoni A Lourida T. The influence of single nucleotide polymorphisms on vitamin D receptor protein levels and function in chronic liver disease. Int J Mol Sci. (2023) 24:11404. doi: 10.3390/ijms241411404, PMID: 37511164 PMC10380285

[37] Triantos C Aggeletopoulou I Kalafateli M Spantidea PI Vourli G Diamantopoulou G. Prognostic significance of vitamin D receptor (VDR) gene polymorphisms in liver cirrhosis. Sci Rep. (2018) 8:14065. doi: 10.1038/s41598-018-32482-3, PMID: 30218108 PMC6138740

[38] Saberi B Dadabhai AS Nanavati J Wang L Shinohara RT Mullin GE. Vitamin D levels do not predict the stage of hepatic fibrosis in patients with non-alcoholic fatty liver disease: a PRISMA compliant systematic review and meta-analysis of pooled data. World J Hepatol. (2018) 10:142–54. doi: 10.4254/wjh.v10.i1.142, PMID: 29399288 PMC5787678

[39] Barchetta I Cimini FA Cavallo MG. Vitamin D and metabolic dysfunction-associated fatty liver disease (MAFLD): an update. Nutrients. (2020) 12:3302. doi: 10.3390/nu12113302, PMID: 33126575 PMC7693133

[40] Evans DM Davey Smith G. Mendelian randomization: new applications in the coming age of hypothesis-free causality. Annu Rev Genomics Hum Genet. (2015) 16:327–50. doi: 10.1146/annurev-genom-090314-050016, PMID: 25939054

[41] Georgakis MK Gill D. Mendelian randomization studies in stroke: exploration of risk factors and drug targets with human genetic data. Stroke. (2021) 52:2992–3003. doi: 10.1161/STROKEAHA.120.032617, PMID: 34399585

[42] Andrews SJ Goate A Anstey KJ. Association between alcohol consumption and Alzheimer’s disease: a Mendelian randomization study. Alzheimers Dement. (2020) 16:345–53. doi: 10.1016/j.jalz.2019.09.086, PMID: 31786126 PMC7057166

[43] Bikle DD Schwartz J. Vitamin D binding protein, Total and free vitamin D levels in different physiological and pathophysiological conditions. Front Endocrinol. (2019) 10:317. doi: 10.3389/fendo.2019.00317, PMID: 31191450 PMC6546814

[44] Bikle DD. The free hormone hypothesis: when, why, and how to measure the free hormone levels to assess vitamin D, thyroid, sex hormone, and cortisol status. JBMR Plus. (2021) 5:e10418. doi: 10.1002/jbm4.10418, PMID: 33553985 PMC7839820

[45] Schwartz JB Gallagher JC Jorde R Berg V Walsh J Eastell R. Determination of free 25(OH) D concentrations and their relationships to Total 25(OH) D in multiple clinical populations. J Clin Endocrinol Metab. (2018) 103:3278–88. doi: 10.1210/jc.2018-00295, PMID: 29955795 PMC6126881

[46] Sheng Q Shi H Liu S Zhuang L Zhao Z Xin Y. Serum 25-hydroxyvitamin D levels and the risk of non-alcoholic fatty liver: a two-sample Mendelian randomization study. Saudi J Gastroenterol. (2023) 29:39–46. doi: 10.4103/sjg.sjg_297_22, PMID: 36254930 PMC10117008

[47] Manousaki D Harroud A Mitchell RE Ross S Forgetta V Timpson NJ. Vitamin D levels and risk of type 1 diabetes: a Mendelian randomization study. PLoS Med. (2021) 18:e1003536. doi: 10.1371/journal.pmed.1003536, PMID: 33630834 PMC7906317

[48] Song J Liu K Chen W Liu B Yang H Lv L. Circulating vitamin D levels and risk of vitiligo: evidence from meta-analysis and two-sample Mendelian randomization. Front Nutr. (2021) 8:782270. doi: 10.3389/fnut.2021.782270, PMID: 35004812 PMC8727691

[49] Zhang Y Jing D Zhou G Xiao Y Shen M Chen X. Evidence of a causal relationship between vitamin D status and risk of psoriasis from the UK biobank study. Front Nutr. (2022) 9:807344. doi: 10.3389/fnut.2022.807344, PMID: 35958262 PMC9359095

