# Supplementary figures and images for: No causal relationship between serum vitamin D levels and alcoholic liver disease: a two-sample bidirectional Mendelian randomization study
Source: Front Nutr. 2024 Jul 31;11:1292954. doi: 10.3389/fnut.2024.1292954 (PMC11322509; doi:10.3389/fnut.2024.1292954)

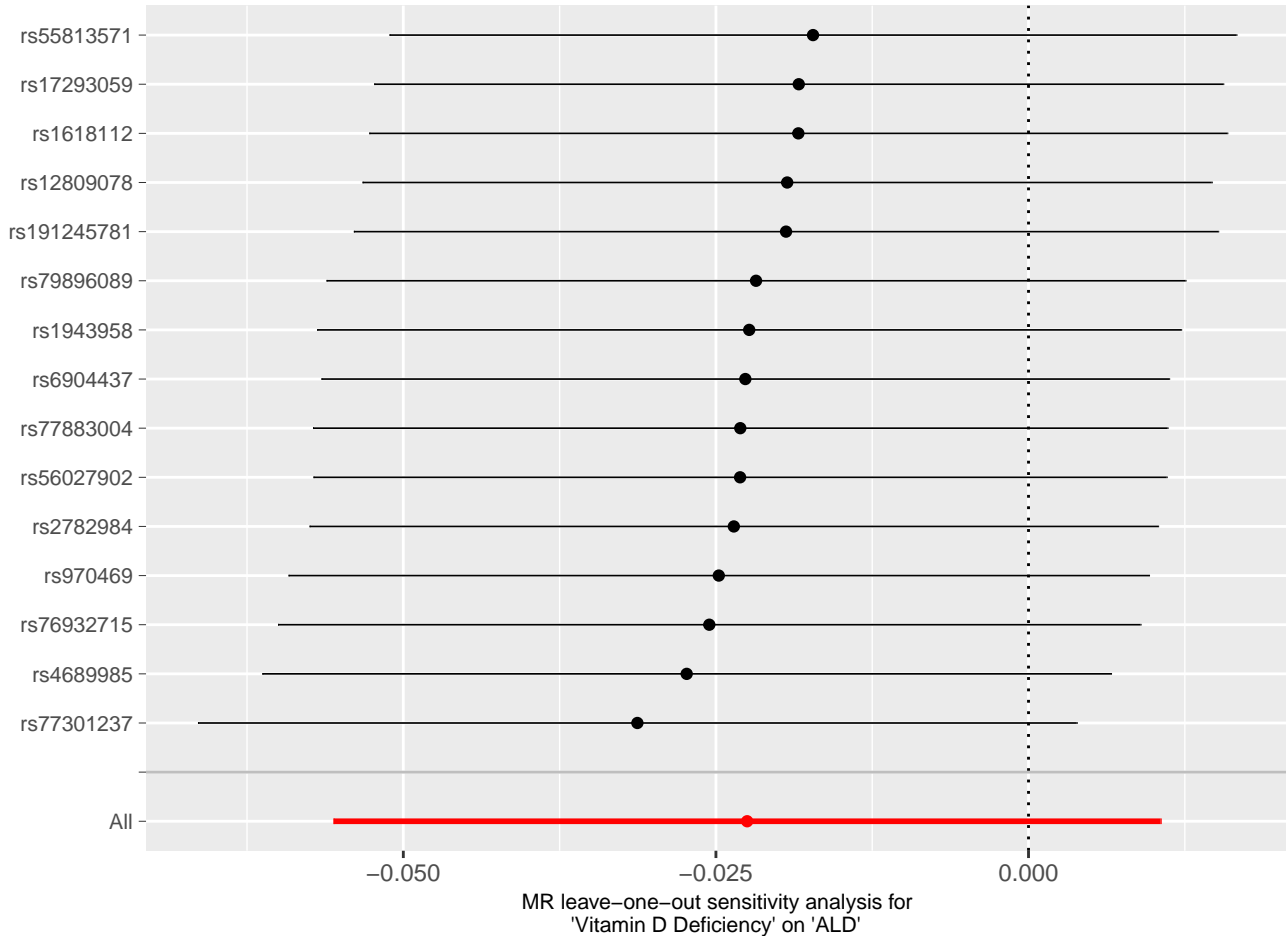

Supplement: Supplementary file 1 [file Data_Sheet_1.ZIP › Supplementary Materials(Vitamin D Deficiency and Alcoholic Liver Disease Mendelian Randomization Study)/forest.pdf]

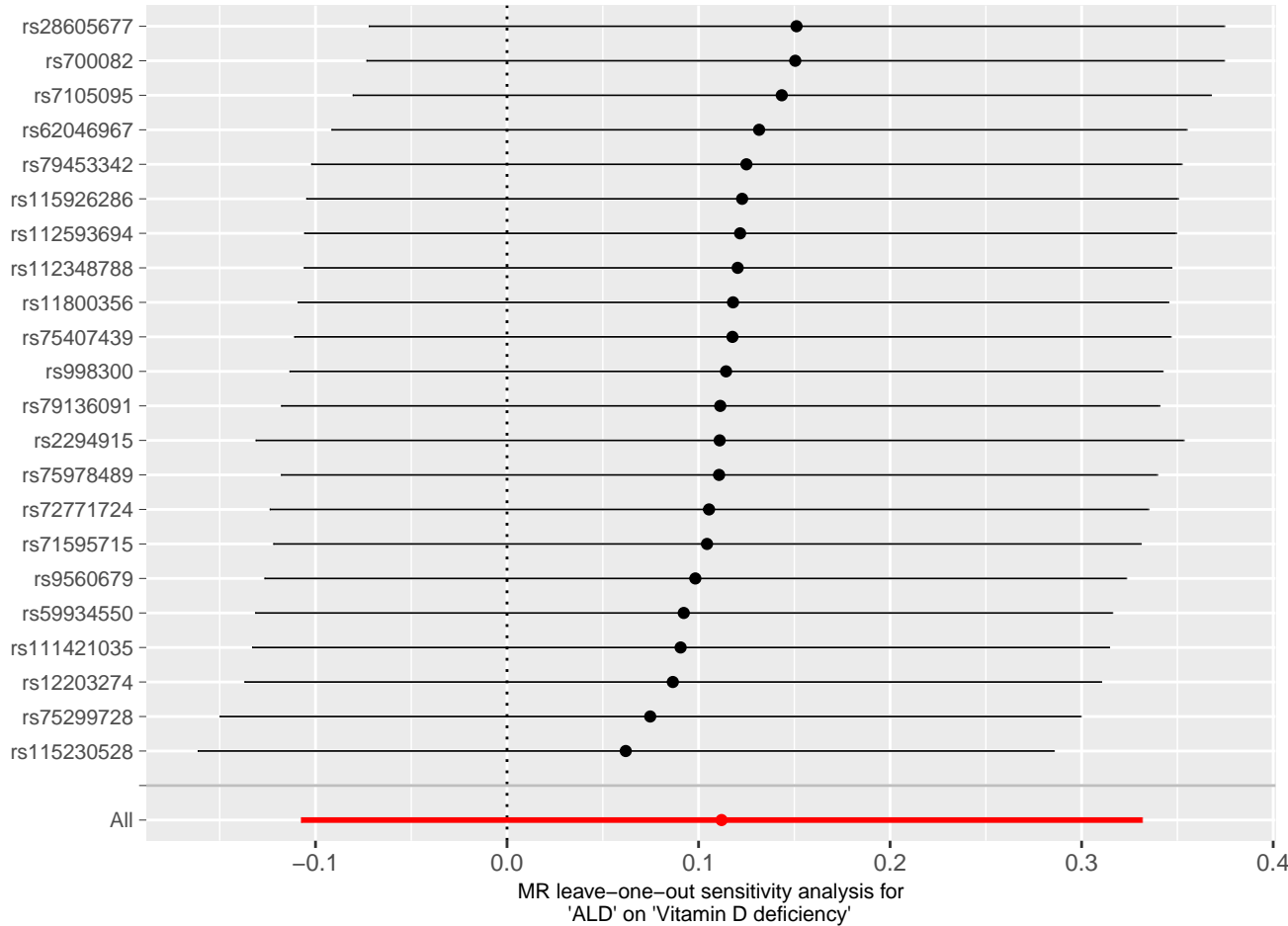

Supplement: Supplementary file 1 [file Data_Sheet_1.ZIP › Supplementary Materials(Vitamin D Deficiency and Alcoholic Liver Disease Mendelian Randomization Study)/forest2.pdf]

# MR Method

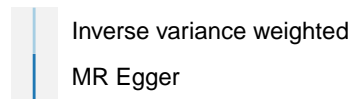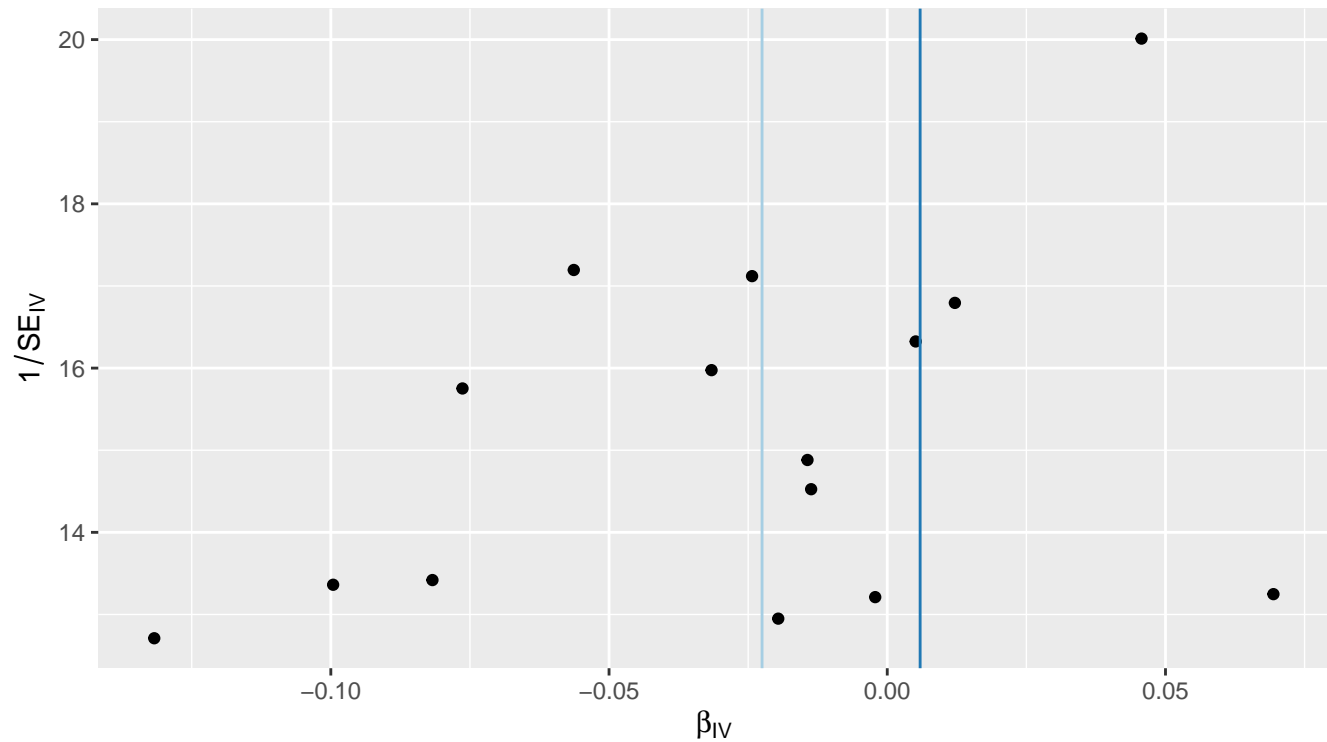

Supplement: Supplementary file 1 [file Data_Sheet_1.ZIP › Supplementary Materials(Vitamin D Deficiency and Alcoholic Liver Disease Mendelian Randomization Study)/funnel.pdf]

# MR Method

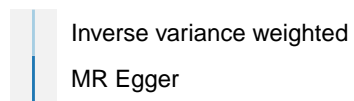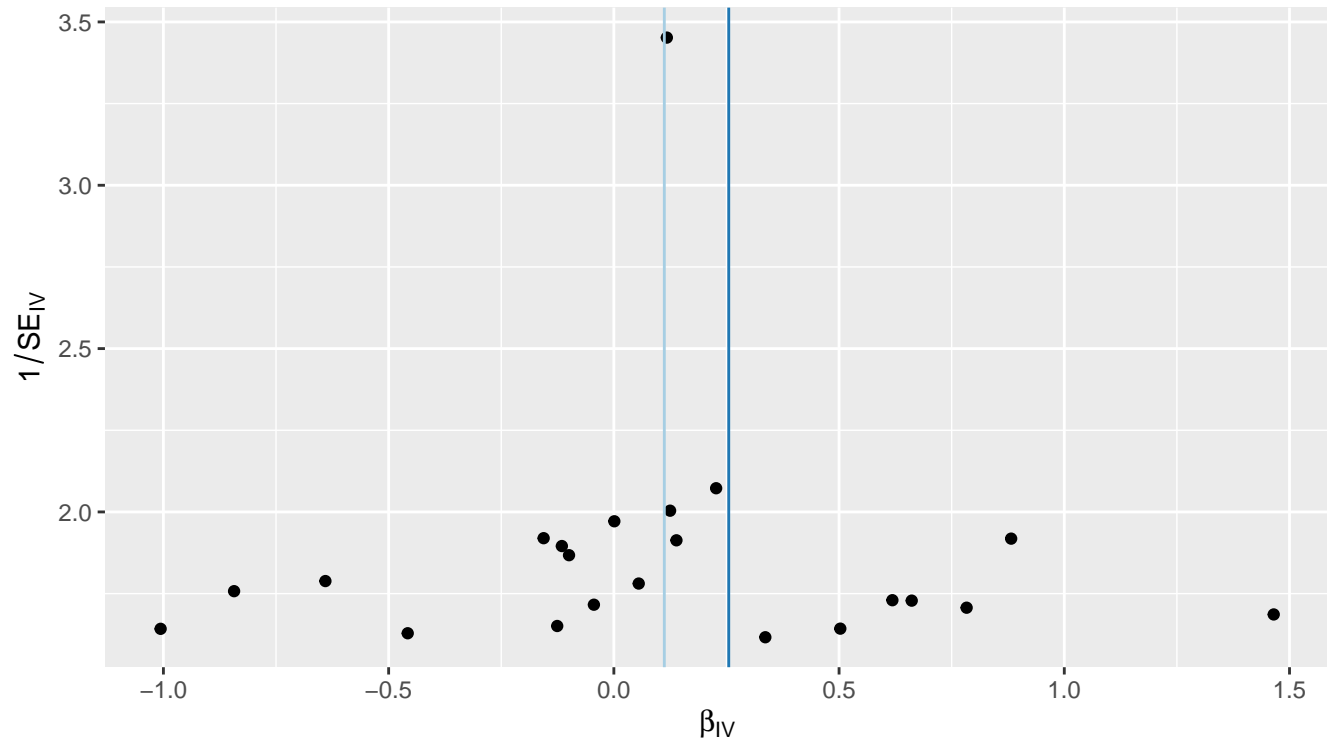

Supplement: Supplementary file 1 [file Data_Sheet_1.ZIP › Supplementary Materials(Vitamin D Deficiency and Alcoholic Liver Disease Mendelian Randomization Study)/funnel2.pdf]

## MR Test

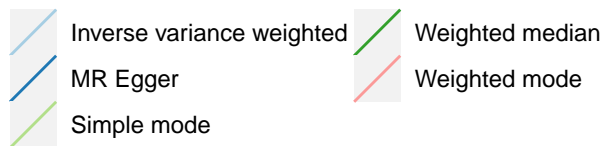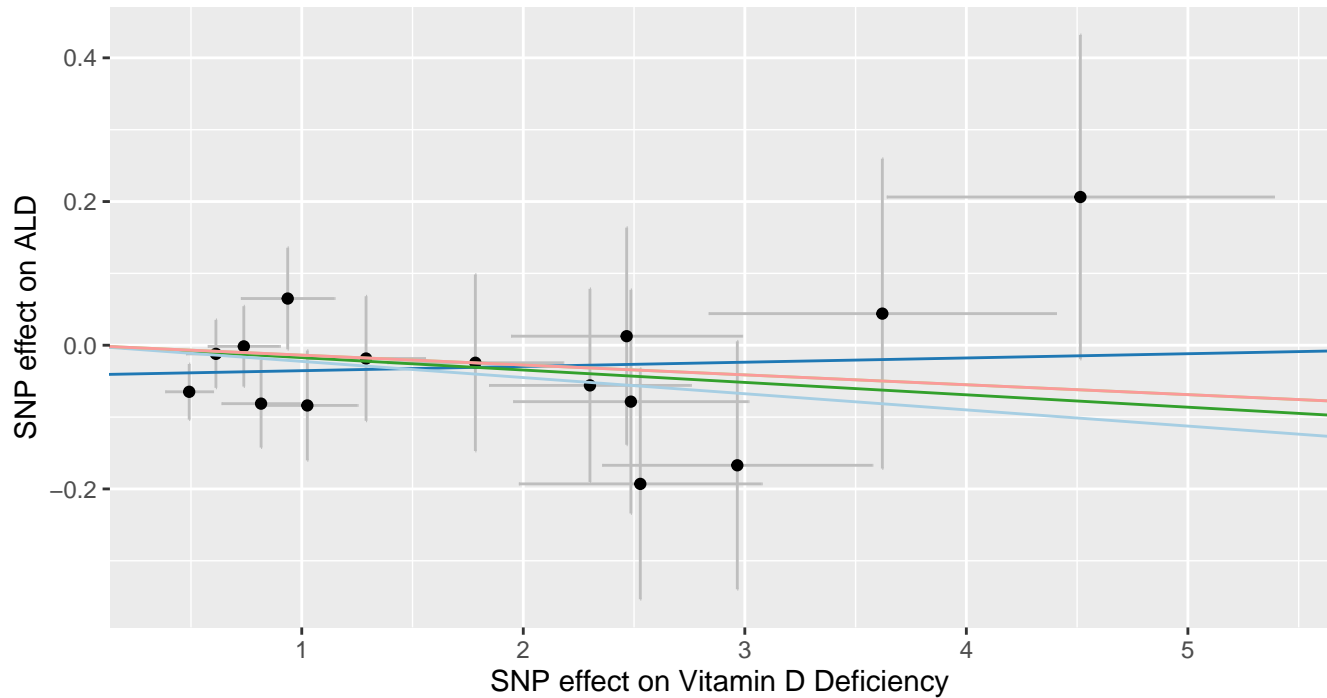

Supplement: Supplementary file 1 [file Data_Sheet_1.ZIP › Supplementary Materials(Vitamin D Deficiency and Alcoholic Liver Disease Mendelian Randomization Study)/scatter.pdf]

## MR Test

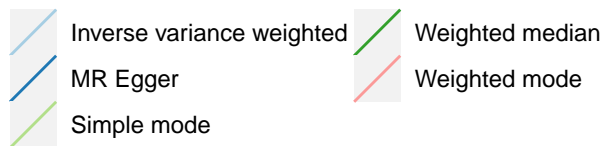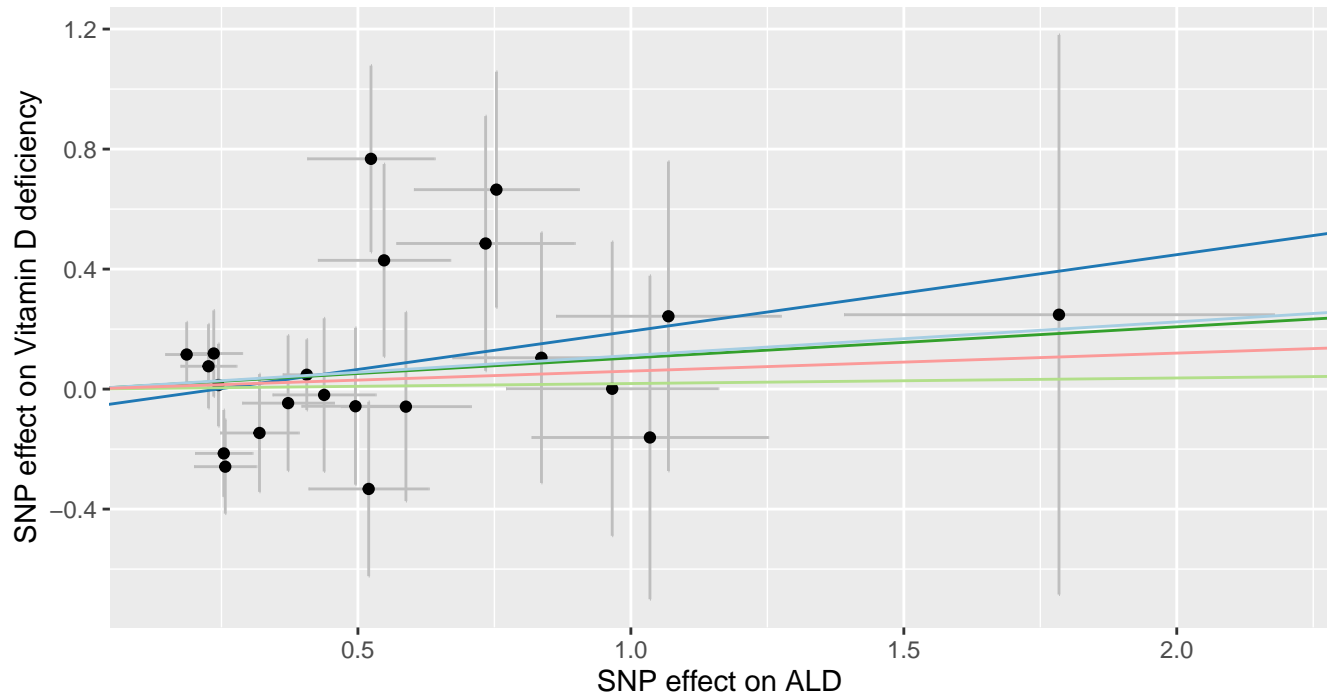

Supplement: Supplementary file 1 [file Data_Sheet_1.ZIP › Supplementary Materials(Vitamin D Deficiency and Alcoholic Liver Disease Mendelian Randomization Study)/scatter2.pdf]
